# Proteomics, toxicity and antivenom neutralization of Sri Lankan and Indian Russell’s viper (*Daboia russelii*) venoms

**DOI:** 10.1590/1678-9199-JVATITD-2020-0177

**Published:** 2021-04-30

**Authors:** Tasnim Faisal, Kae Yi Tan, Nget Hong Tan, Si Mui Sim, Christeine Ariaranee Gnanathasan, Choo Hock Tan

**Affiliations:** 1 Department of Pharmacology, Faculty of Medicine, University of Malaya, Kuala Lumpur, Malaysia.; 2 Department of Molecular Medicine, Faculty of Medicine, University of Malaya, Kuala Lumpur, Malaysia.; 3 Department of Clinical Medicine, Faculty of Medicine, University of Colombo, Colombo, Sri Lanka.

**Keywords:** Geographical variation, Venomics, Antivenomics, Antivenom potency

## Abstract

**Background:**

The western Russell’s viper (*Daboia russelii*) is widely distributed in South Asia, and geographical venom variation is anticipated among distant populations. Antivenoms used for Russell’s viper envenomation are, however, raised typically against snakes from Southern India. The present study investigated and compared the venom proteomes of *D. russelii* from Sri Lanka (DrSL) and India (DrI), the immunorecognition of Indian VINS Polyvalent Antivenom (VPAV) and its efficacy in neutralizing the venom toxicity.

**Methods:**

The venoms of DrSL and DrI were decomplexed with C_18_ high-performance liquid chromatography and SDS-polyacrylamide gel electrophoresis under reducing conditions. The proteins fractionated were identified through nano-ESI-liquid chromatography-tandem mass spectrometry (LCMS/MS). The immunological studies were conducted with enzyme-linked immunosorbent assay. The neutralization of the venom procoagulant effect was evaluated in citrated human plasma. The neutralization of the venom lethality was assessed *in vivo* in mice adopting the WHO protocol.

**Results:**

DrSL and DrI venom proteomes showed comparable major protein families, with phospholipases A_2_ (PLA_2_) being the most abundant (> 60% of total venom proteins) and diverse (six protein forms identified). Both venoms were highly procoagulant and lethal (intravenous median lethal dose in mice, LD_50_ = 0.24 and 0.32 µg/g, for DrSL and DrI, respectively), while lacking hemorrhagic and anticoagulant activities. VPAV was immunoreactive toward DrSL and DrI venoms, indicating conserved protein antigenicity in the venoms. The high molecular weight venom proteins were, however, more effectively immunorecognized than small ones. VPAV was able to neutralize the coagulopathic and lethal effects of the venoms moderately.

**Conclusion:**

Considering that a large amount of venom can be injected by Russell’s viper during envenomation, the potency of antivenom can be further improved for optimal neutralization and effective treatment. Region-specific venoms and key toxins may be incorporated into the immunization procedure during antivenom production.

## Background

Snakebite envenomation is an important public health threat that has been instated as a neglected tropical disease in 2017 by the World Health Organization (WHO) [1]. The disease burden of snakebite envenomation, however, continues to affect millions of impoverished populations to a great extent. The victims are typically people whose livelihoods are dependent on agriculture activities and have poor access to appropriate medical care for snakebite [2,3]. Indeed, snakebite envenomation has been aptly called the disease of poverty [4,5], although the epidemiology of snakebite remains lacking in many parts of the world. Global estimates indicated that there are 4.5-5.4 million people bitten by snakes a year, and that 1.8-2.7 millions of them develop clinical toxicity (snakebite envenomation) with a death toll that ranges from 81,000 to 138,000 [3,6]. Based on more recent community-based studies [7-10], there is clear evidence that South Asia is one of the most heavily affected regions. In India alone, there were 2.8 million reported cases of snakebite annually with 46,900 deaths [7]. The neighboring country of Sri Lanka also shares a similar burden of snake envenomation, with approximately 80,000 snakebite cases reported and 400 deaths annually [8].

In Sri Lanka and India, the “Big Four” (Russell’s viper, spectacled cobra, common Indian krait and saw-scaled viper) represent the four most common and medically important venomous snake species that cause snakebite envenomation [2,3]. However, widely distributed venomous species such as Russell’s viper, are known to exhibit geographical variations in its venom composition [11-15]. Indeed, the composition of snake venom is influenced by various ecological factors; thus, it can vary significantly across the geographical distribution of the species as the snakes adapt to distinct habitats. The geographical variation of Russell’s viper venom has been attributed as the cause of differences in the clinical features of envenomation reported in various regions [16].

*Daboia russelii* or the western Russell’s viper is widespread throughout South Asia, including Pakistan, India, Bangladesh, Nepal, Sri Lanka, Bhutan [17], Jammu and Kashmir [18]. The snake is aggressive, ready to strike when provoked, and able to deliver a large amount of lethal venom with each bite. In view of its wide distribution and the high fatality of envenomation, *D. russelii* is considered as one of the deadliest snakes in South Asia [19,20]. The major manifestation of Russell’s viper envenomation is hemostatic derangement that results in coagulopathy, internal hemorrhage, hypovolemic shock and major organ failure [21]. Besides causing hemotoxicity, Russell’s viper envenoming in different geographical areas has also been reported to cause neurotoxicity [22], capillary leakage syndrome [23,24], myotoxicity and acute kidney injury [19,20,25,26] as well as pituitary infarction [16,27]. Apparently, the clinical manifestations of Russell’s viper envenomation vary due to intra-species geographical variation in the venom composition. The geographical variation of venom also affects the efficacy of envenomation treatment, since the venom immunogen used in antivenom production was sourced from limited populations of Russell’s viper in Southern India.

Hence, a comprehensive study of the geographical variation of snake venom is crucial. The advent of proteomics in recent years has greatly advanced the composition study of snake venom from diverse species [28,29]. A number of studies have reported the venom proteomes of *D. russelii* from various locales including Sri Lanka [13,30], Pakistan [11-13], Bangladesh [13] and the western (Haffkine Institute, Mumbai) [15], eastern (Burdwan and Nadia) [14] and southern (Irula Snake Catchers Society, ISCC) [13,31] regions of India, using different proteomic platforms and quantitation approaches. These studies, collectively, demonstrated geographical variation in the venoms of Russell’s viper from different locales. However, it is challenging to compare the proteomes, toxicity and neutralization of the venom samples from various locales when these studies adopted different experimental approaches. In some studies, data of protein subtypes/proteoforms and relative abundances were incomplete or unavailable. A good phylovenomic model was recently reported for the Russell’s vipers from India, Sri Lanka, Pakistan and Bangladesh by Pla *et al*. [13], demonstrating that a standardized analytical approach is useful when investigating the venom variation. Following the proteomic study, it is also important to examine the neutralization activity of antivenom against all venom samples, so that the suitability of an antivenom for use in a particular region can be ascertained.

Although venom-induced toxicity can be circumvented by supportive treatment, antivenom remains the only definitive and effective treatment for snakebite envenomation [32]. In India, the antivenom products are usually formulated as polyvalent antivenoms against the “Big Four” species (*Daboia russelii*, *Naja naja*, *Bungarus caeruleus*, and *Echis carinatus*), produced by a few manufacturers. Of these, the Indian VINS polyvalent antivenom (VPAV) is one of the most commonly sourced products, which is also exported to neighboring countries such as Sri Lanka and Pakistan to meet the high local need for antivenom [33,34]. The Indian antivenom is raised against the “Big Four” of Indian origin, typically sourced from the Irula district of Tamil Nadu in South India. Thus, it is necessary to rigorously examine how effective the Indian antivenom is in neutralizing the toxic effects of the Sri Lankan *D. russelii* venom, besides comparing the venom compositions and immunological profiles between the two geographically separated populations of *D. russelii*. In this study, we characterized the venom proteome of Russell’s viper originated from the northern, western and south-western regions of Sri Lanka. These were snakes implicated in real snakebite envenomation, brought to the hospital and authenticated by the herpetologist at the Snake Venom Research Laboratory & Herpetarium (SVRL&H), Faculty of Medicine, University of Colombo, Sri Lanka. The proteomic study was followed by an investigation of the immunoreactivity and neutralization capacity of VPAV, a widely used Indian antivenom product in the country. For comparison, the experiments were conducted in parallel with the Russell’s viper venom of South Indian origin, which is used in the production of the Indian antivenom.

## Materials and methods

### Materials

Ammonium bicarbonate, iodoacetamide and dithiothreitol (DTT) were purchased from Sigma-Aldrich (USA). HPLC grade solvents, mass-spectrometry grade trypsin protease and Spectra™ Multicolor Broad Range Protein Ladder (10-260 kDa) were purchased from Thermo Scientific™ (USA). ExcelBand™ 3 color High Range Protein Marker (10-245 kDa) was purchased from SMOBIO (Taiwan). Millipore ZipTip® C18 Pipette Tips were obtained from Merck (USA). Activated partial prothrombin time (APTT) and prothrombin time (PT) reagents were purchased from R^2^ Diagnostics (Swansea, UK). Other chemicals and reagents of analytical grade were purchased from Sigma-Aldrich (USA).

### Venoms and antivenom

*Daboia russelii* venom of Sri Lanka was milked from multiple specimens originated from Anuradhapura (Northern region), Colombo (Western region), Ratnapura and Galle (South-western region) of the island country. The Indian sample of *D. russelii* venom was from Tamil Nadu region (South India) for comparative study. The lyophilized venom samples were stored at ‒20 °C until use. Indian VINS Polyvalent Antivenom (VPAV, Batch no:01AS12041) is the antivenom used in the current study. The antivenom was produced from the antisera of horses hyperimmunized against the venom of Indian “Big Four”, *i.e.*, *D. russelii, N. naja, B. caeruleus* and *E. carinatus*. The antivenom was reconstituted in 10 mL ultrapure water as per the manufacturer’s instruction prior to use.

### Animal supply

Albino mice of ICR strain (20−25 g) were supplied by the Animal Experimental Unit, Faculty of Medicine, University of Malaya. The protocol was approved by the Institutional Animal Care and Use Committee of the Faculty of Medicine, University of Malaya (Reference number: 20140911/PHAR/R/TCH) and carried out based on the Council for International Organizations of Medical Sciences (CIOMS) guidelines on animal experimentation [35].

### Determination of antivenom protein concentration

Antivenom protein concentration (VPAV) was determined using bicinchoninic acid (BCA) protein assay kit (Thermo Scientific™, USA), with bovine serum albumin (BSA) used as the protein standard calibrator. The antivenom concentration was expressed as mean ± S.E.M. of triplicates.

### C_18_ reverse-phase HPLC of the Sri Lankan and Indian *D. russelii* venom

Three milligrams of lyophilized venoms were reconstituted in 0.1% TFA and fractionated by LiChrospher^®^ WP300 C_18_ column (100 Å, 250 mm x 4.6 mm) using a Shimadzu LC-20AD HPLC system (Shimadzu, Co., Ltd. Japan). The proteins were eluted at a flow rate of 1 mL/min [36] by Solvent B (0.1% TFA in 100% acetonitrile) with a linear gradient of 0-5% (10 min), followed by 5-30% (20 min), 30-55% (120 min) and 50-70% (20 min). Protein elution was monitored at 215 nm, manually collected, pooled and subjected to SDS-PAGE and in-solution tryptic digestion. The digested peptides were then individually analyzed by shotgun proteomic analysis.

### SDS-PAGE of venom and eluted HPLC fractions

Whole venoms (50 µg) and HPLC-eluted venom fractions were electrophoresed with 15% SDS-PAGE gel under reducing conditions at 90 V for 2 h, according to Laemmli [37]. Proteins in the gel were stained using Coomassie Brilliant Blue R-250. The relative intensity of protein gel bands were determined using myImage Analysis software (Thermo Scientific™, USA).

### Nano-electrospray-ionization-liquid chromatography tandem mass spectrometry (nano-ESI-LCMS/MS)

The protein fractions obtained from RP-HPLC were reduced by DTT, alkylated by iodoacetamide, and digested by trypsin as previously described [38]. The trypsin-digested peptides were then desalted and extracted with Millipore Zip Tip and subjected to nano-ESI-LCMS/MS analysis, using an Agilent 1260 Infinity Nanoflow LC system (Agilent, USA) coupled to the Accurate-Mass Q-TOF 6550 series. The samples were reconstituted in 7 µL of 0.1% formic acid in water and loaded onto HPLC Large-Capacity Chip Column Zorbax 300-SB-C18 (160 nL enrichment column, 75 µm x 150 mm analytical column and 5 µm particles) (Agilent, USA). One µL of the sample was injected and eluted at a flow rate of 0.4 µL/min, with a linear gradient of 5-7% of solvent B (0.1% formic acid in 100% acetonitrile). The drying gas flow used was 11 L/min and the drying has temperature was 290 °C. Fragmentor voltage was set to 175 V and capillary voltage was set to 1800 V. Mass spectra were acquired using Mass Hunter acquisition software (Agilent, USA). Data with MH^+^ mass range between 50 and 3000 Da were collected and processed with Agilent Spectrum Mill MS Proteomics Workbench software package version B.04.00 against a merged database incorporating non-redundant NCBI database of Serpentes (taxid: 8570) and our in-house venom gland transcript database as previously described [39,40]. Carbamidomethylation and oxidized methionine were specified as a fixed modification and a variable modification, respectively. For protein validation, the following filtering parameters were applied: protein score > 20, peptides score > 10 and scored peak intensity (SPI) > 70%. Identified proteins were further filtered to achieve a false discovery rate (FDR) < 1%, and only proteins with at least 2 “Distinct Peptides” matched were considered significant for identification. 

The relative abundance of protein (X) eluted in chromatographic fraction (Y) was estimated based on the following formula as previously reported [36]:

$$\begin{array}{ccc} Protein\;X\;abundance\;in\;Fraction\;Y\;= & \frac{Mean\;spectral\;intensity\;of\;protein\;X\;in\;Fraction\;Y}{Total\;mean\;spectral\;intensity\;of\;all\;proteins\;in\;Fraction\;Y} & \times \;Area\;under\;the\;curve\;of\;Fraction\;Y\;(\%) \end{array}$$

### Data availability

The mass spectrometry data of proteomics were deposited to the ProteomeXchange Consortium (http://proteomecentral.proteomexchange.org/) via the iProX partner repository [41], with the dataset identifier PXD019981. 

### Immunological binding assay of antivenom

The immunological binding activities of antivenom and venom antigens were examined using indirect enzyme-linked immunosorbent assay (ELISA) [42]. In brief, the immunoplate wells were pre-coated with 10 ng venoms from D. russelii (Sri Lankan and Indian samples) and C. rhodostoma (Malaysian origin, used as negative control) overnight at 4 °C. The venoms were then discarded, the wells were flicked dried and washed four times with phosphate-buffered saline containing 0.5% Tween®20 (PBST). Antivenom (VPAV) with a stock protein concentration of 20 mg/mL was prepared and serially diluted (1:300 to 1:24300) in PBST. The antivenom preparations (100 µL) were added into the venom-coated wells and incubated for 1 h at room temperature. The wells were washed four times with PBST, and horseradish peroxidase-conjugated anti-horse-IgG (Jackson ImmunoResearch Inc, USA) (1:8000 dilution) were then added into the wells. The wells were incubated for 1 h and then washed again before adding 100 µL substrate (0.5 mg/mL o-phenylenediamine and 0.006% hydrogen peroxide in 0.1 M citrate-phosphate buffer, pH 5.0). The plate was left at room temperature in the dark for 30 min. The reaction was terminated by adding 50 µL of 12% sulfuric acid and the absorbance was read at 495 nm using Tecan M1000Pro Multimode plate reader (TECAN, Switzerland). The activity of venom-antivenom immunological binding was measured in absorbance unit, expressed in mean ± S.E.M. of triplicate experiments. The half maximal concentration (EC_50_) for the antivenom binding activity was defined as the concentration of antivenom that corresponding to half of the maximal absorbance.

### *ELISA immunoprofiling of D. russelii venoms*

The venom protein fractions eluted from reverse-phase HPLC were freeze-dried and subsequently reconstituted in ultrapure water. Ten ng of venom proteins from each eluted fraction were coated on the immunoplates, followed by overnight incubation at 4 °C. Indirect ELISA was performed as outlined above with an optimized VPAV antivenom dilution of 1:2700.

### Determination of procoagulant activity and neutralization by antivenom

The procoagulant activity of venom was determined using human citrated plasma as substrate [12]. Fresh blood was collected into sodium citrate tubes and centrifuged at 1500 rpm for 12 min. Plasma was then aliquoted for assay use. Venom samples (100 µL, diluted in phosphate buffer saline to various concentrations) were added into 96-microplate wells. Following this, the citrated human plasma (100 µL) containing 40 µL 0.4 M calcium chloride (CaCl_2_)/mL was added simultaneously to the wells that contained venom samples. Coagulation activity was measured using the turbidimetric method as previously described [43], where the formulation of clots was real-time monitored at 405 nm absorbance at 37 °C for 15 minutes, using the Tecan M1000Pro Multimode plate reader (TECAN, Switzerland). The time for plasma clotting was taken as the time when the absorbance reading was 0.02 U greater than the mean of the first two absorbance measurement. The minimum coagulation dose (MCD) is defined as the venom concentration that induces substrate coagulation in 3 min.

In neutralization assay, the venom samples at a dose of 2 MCD each were pre-incubated with various dilutions of VPAV at 37 ^o^C for 30 min. The total volume of venom-antivenom mixture was standardized at 50 µL. A hundred microliters of citrated human plasma (premixed with 40 µL 0.4 M CaCl_2_/mL) was then added simultaneously into the wells that contained the venom-antivenom mixtures. The clotting time was then determined as described above. The effective dose (ED) of antivenom was defined as the dose of antivenom in volume (µL) that prolonged the clotting time of the citrated human plasma to three times that of the control (2 MCD of venom, without antivenom). The efficacy was also expressed as an effective ratio in terms of venom amount (µg) neutralized per microliter of antivenom (µL) at the point corresponding to ED, using the following formula:

$$\begin{array}{cc} Effective\;Ratio\;\left(ER\right)= & \frac{2\;minimal\;coagulation\;dose\;\left(MCD,\;\;\mu g\right)}{Antivenom\;volume\;that\;prolonged\;clotting\;time\;3\;times\;that\;of\;control\;\left(\mu L\right)} \end{array}$$

### Determination of venom anticoagulant activity

Fresh blood was collected into sodium citrate tubes and centrifuged at 1500 rpm for 12 min. Various dilutions of venoms were prepared in a final volume 15 uL and loaded into 96-microplate well plate. One hundred fifty uL of pre-warmed plasma at 37 °C was added into each well and incubated at 37°C for 2 min. Twenty uL of 0.25 M CaCl_2_ was added to each well to induce clotting. Clotting time was recorded (milliseconds/seconds). For negative control, plasma (150 μL) was incubated with PBS (15 uL). An extrinsic and intrinsic assay using APTT, and PT reagents were carried out with a modified method [44]. Anticoagulant activity of venom was determined based on its prolongation of negative control clotting time by 1 s.

### Determination of venom lethality and neutralization by antivenom

A hundred microliters of venom prepared at varying concentrations was injected intravenously (via the caudal vein) into mice, as previously described [12]. The mice were allowed access to food and water ad libitum. The survival ratio of the mice was recorded after 24 h and the LD_50_ was calculated using the Probit analysis method [45].

The potency of antivenom in neutralizing the lethality of venom was determined using an immunocomplexation method as described previously [42]. A challenge dose of the venom (5 LD_50_) was preincubated with various dilutions of VPAV at 37 °C for 30 min. The incubation mixture was then injected intravenously into the mice and the survival ratio of the mice was recorded after 24 h. The median effective dose (ED_50_) of antivenom was expressed as the volume of antivenom in μL at which 50% of the challenged animals survived. Effective ratio (ER_50_) was derived from ED_50_ and expressed as the ratio of venom to antivenom (mg/mL) at which 50% of the challenged animals survived. The neutralization potency (P) of antivenom is defined as the amount of venom completely neutralized per unit volume of antivenom (mg/mL) was calculated accordingly [46]. The P (potency) value was then divided by the protein concentration of antivenom to obtain the “normalized potency” (n-P), defined as the amount of venom in mg completely neutralized per unit mass of antivenom proteins in gram [47]. 

### Statistical analysis

Statistical significance of bioassay was analyzed with student t-test. In in vivo studies, the LD_50_ of the venom and ED_50_ of antivenom were expressed as means with 95% confidence interval (CI). The values were calculated using the Probit analysis method of Finney [45] with BioStat 2009 analysis software (AnalystSoft Inc., Canada).

## Results

### *Protein decomplexation and identification of D. russelii venoms*

**Figure 1:**
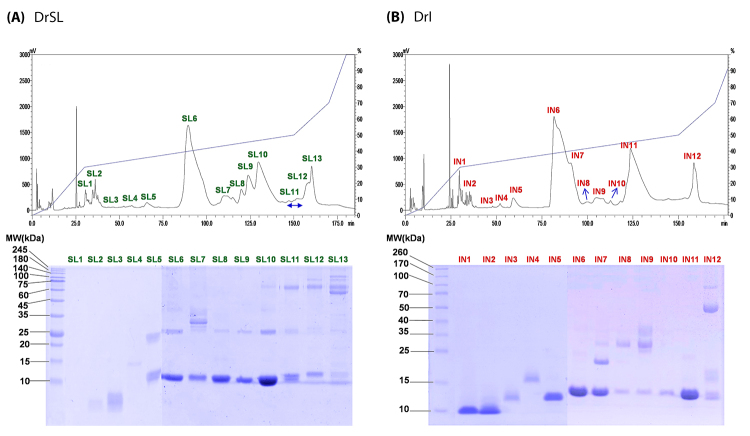
Protein decomplexation of snake venom: **(A)** DrSL, Sri Lankan Daboia russelii; **(B)** DrI, South Indian Daboia russelii. Upper panel: C_18_ reverse-phase high-performance liquid chromatography (RP-HPLC) of Daboia russelii venoms (3 mg). Lower panel: 15% SDS-PAGE of the venom fractions under reducing conditions.

Reverse-phase HPLC of Sri Lankan and South Indian D. russelii venoms yielded comparable chromatogram profiles, with 13 and 12 fractions eluted, respectively (Figure 1A, upper panel). SDS-PAGE of the fractions under reducing conditions showed that the venoms were composed of proteins of diverse molecular masses ranging from less than 10 kDa to 140 kDa (Figure 1B, lower panel). Proteins identified within the respective fractions by nano-ESI-LCMS/MS were shown in Additional file 1 (Sri Lankan D. russelii, DrSL) and Additional file 2 (Indian D. russelii, DrI). The mass spectrometry data for each protein fraction analyzed were provided in Additional file 3.

**Figure 2:**
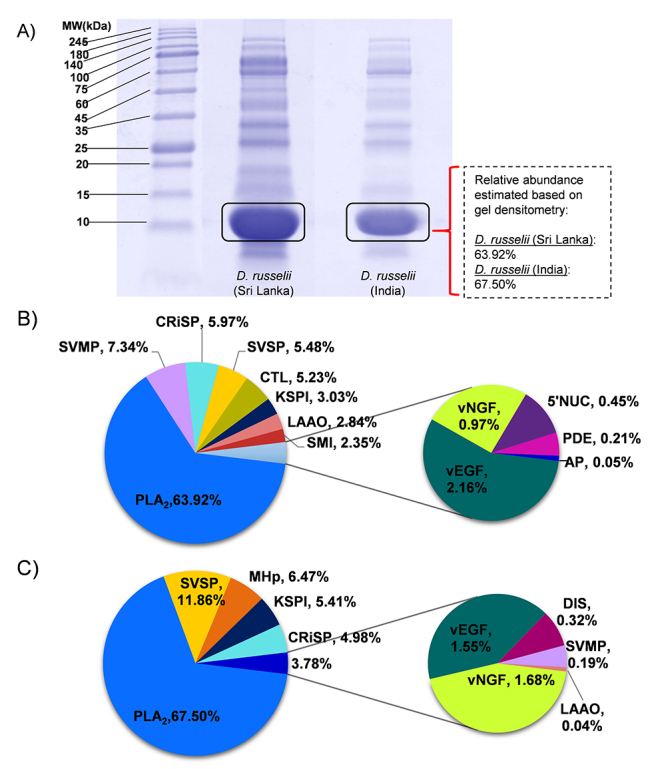
**(A)** Sodium dodecyl sulfate-polyacrylamide gel of Sri Lankan (left) and South Indian (right) *D. russelii* whole venoms, under reducing conditions. Gel intensity was measured by densitometry with the aid of myImageAnalysis software. **(B)** Venom proteome with relative protein abundance of Sri Lankan Daboia russelii venom. **(C)** Venom proteome with relative protein abundance of South Indian Daboia russelii venoms. PLA_2_: phospholipase A_2_; SVMP: snake venom metalloproteinase; SVSP: snake venom serine protease; KSPI: Kunitz-type serine protease inhibitor; CRiSP: cysteine-rich secretory protein, SMI: snake venom metalloproteinase inhibitor; vNGF: nerve growth factor; vEGF: venom endothelial growth factor; Snaclec: snake venom C-type lectin/lectin-like proteins; PDE: phosphodiesterase; LAAO: L-amino acid oxidase; 5′-NUC: 5′-nucleotidase; MHp: moderate to high molecular weight proteins.

The venom proteins detected, and their relative abundances were shown and compared between DrSL and DrI in Table 1. LCMS/MS analysis of the digested peptides and data mining categorized the venom proteins into 13 (DrSL) and 9 (DrI) protein families. Based on the HPLC chromatograms and SDS-PAGE, a distinct major protein band of approximately 13 kDa was found in the HPLC fractions SL6 to SL10 (DrSL) and fractions IN6, IN7 and IN11 (DrI), as shown in Figure 1. These proteins corresponded to phospholipases A_2_ (PLA_2_) which accounted for > 60% of the total venom proteins, estimated based on the chromatographic peak area of the venoms (Figure 1) and the densitometry of venom SDS-PAGE (Figure 2A). Indeed, quantitation by mass spectrometry analysis showed that the PLA_2_ dominated both venom proteomes with a relative abundance of 63.92% (DrSL) and 67.5% (DrI) (Figure 2B), supporting that this enzymatic toxin family constituted the main bulk of proteins in both of the Russell’s viper venoms. Other major protein components included snake venom metalloproteinase (SVMP) at 7.34% for DrSL, and snake venom serine protease (SVSP) at 11.86% for DrI (Figure 2B). In comparison, DrI had a much higher abundance of SVSP (11.86%) than DrSL (5.48%), while the SVMP detected in DrI venom was much lower compared with DrSL. The smaller proteins of snake venom metalloproteinase inhibitor (SMI) and snaclec/C-type lectin/lectin-like proteins (CTL) were present in DrSL venom at 2.35% and 5.23% abundances, respectively, but undetected in DrI venom proteome. Both venoms contained similar abundances of cysteine-rich secretory protein (CRiSP, 5-6%), venom nerve growth factor (vNGF, 1-2%) and venom endothelial growth factor (vEGF, 1-2%). DrSL venom contained high molecular weight proteins such as L-amino acid oxidase (LAAO) at 2.84% abundance, whereas 5’nucleotidase (5’NUC) and phosphodiesterase (PDE) were found at trace amounts (< 0.5%). On the other hand, the LAAO content in DrI venom was much lower (< 0.1%), while 5’NUC and PDE were undetected. A trace amount of aminopeptidase (0.05%, AP) too was detected in DrSL venom. A small content of PLA_2_ (~3%) was also eluted in DrI HPLC fraction 12 (IN12). In addition, SDS-PAGE of the fraction revealed 3 gel bands with low intensity (approximately 6% of protein abundance), ranging from moderate to high molecular weights. These proteins (labeled MHp) were not identifiable with repeated analysis of tandem mass spectrometry. Comparison with venom fraction SL13 of DrSL (also eluted at 150 min, Figure 1A) suggested that the MHp proteins in IN12 fraction were likely isoforms of SVMP, 5-NUC’, PDE and/or LAAO (Additional file 2). 

**Table 1:** Overview of major protein families of Daboia russelii venoms from Sri Lanka, India and Pakistan examined under the same experimental conditions.

>Protein family/major protein subfamilies*	Relative abundance (%)		
	Sri Lanka (multiple locales)	India (South)	Pakistan (Sindh Delta)**
**Phospholipase A_2_ (PLA_2_)**	**63.92%**	**67.50%**	**63.76%**
Basic phospholipase A_2_	63.92%	64.36%	41.85%
Acidic phospholipase A_2_	-	3.14%	21.92%
Neutral Phospholipase A_2_	-	-	-
**Snake venom metalloproteinase (SVMP)**	**7.34%**	**0.19%**	**2.52%**
**Class PII**	***0.19%***	-	-
P-IIa sub-subfamily	0.05%	-	-
P-IIb sub-subfamily	0.14%	-	-
**Class PIII**	***7.15%***	***0.19%***	***2.52%***
P-IIIa sub-subfamily	3.74%	-	-
P-IIIc sub-subfamily	0.82%	-	-
P-IIId sub-subfamily	2.59%	0.19%	2.52%
**Snake venom serine protease (SVSP)**	**5.48%**	**11.86%**	**5.5%**
**Kunitz-type serine protease inhibitor (KSPI)**	**3.03%**	**5.41%**	**16.0%**
**Snaclec, C-type lectin & C-type lectin-like protein (CTL)**	**5.23%**	-	**1.3%**
**Cystein-rich secretory protein (CRiSP)**	**5.97%**	**4.98%**	**1.3%**
**L-amino acid oxidase (LAAO)**	**2.84%**	**0.04%**	**0.8%**
**Snake venom metalloproteinase inhibitor (SMI)**	**2.35%**	-	-
**Venom endothelial growth factor (vEGF)**	**2.16%**	**1.55%**	**4.3%**
**Venom nerve growth factor (vNGF)**	**0.97%**	**1.68%**	**1.1%**
**Nucleotidase (5' NUC)**	**0.45%**	-	**0.1%**
**Phosphodiesterase (PDE)**	**0.21%**	-	**2.5%**
**Disintegrin (DIS)**	-	**0.32%**	-
**Moderate to high molecular weight proteins (MHp)**	-	**6.47%**	-
**Uncharacterized protein (UP)**	-	-	**0.7%**
**Aminopeptidase (AP)**	**0.05%**	-	-

*Protein names based on sequence similarity in UniProt database through BLAST search.

**The values were according to Faisalet al. [12] reported by the same laboratory.

### Antivenom protein concentration

The protein concentration of reconstituted VINS Polyvalent Antivenom (VPAV) was determined to be 84.93 ± 4.3 mg/mL.

### *Immunoreactivity of antivenom toward D. russelii venoms*

**Figure 3:**
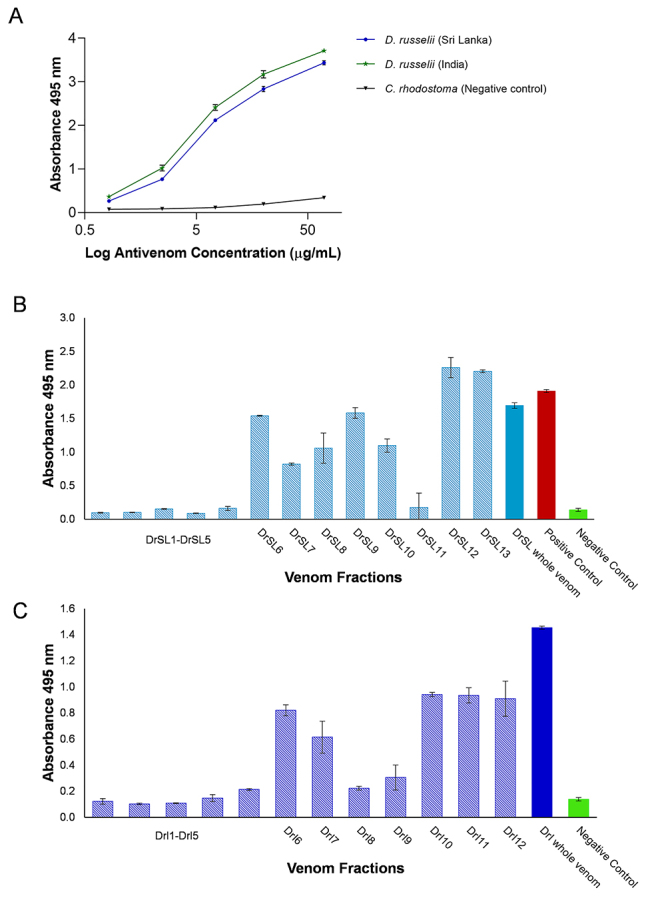
Immunological binding activity of the VPAV with **(A)** whole venoms of the Sri Lankan and South Indian *Daboia russelii*; **(B)** protein fractions of the venom eluted by reverse-phase HPLC (DrSL); **(C)** protein fractions of the venom eluted by reverse-phase HPLC (DrI). Indian D. russelii venom was used as positive control and Calloselasma rhodostoma venom as negative control. Absorbance values were obtained by indirect ELISA and expressed as mean ± S.D. from three experiments.

The immunological binding activity of VINS Polyvalent Antivenom (VPAV) increased dose-dependently toward both DrSL and DrI venoms (Figure 3A). Between the two venoms tested, the antivenom immunoreactivity binding activity was markedly higher toward DrI sample, as shown by a significantly lower half maximal effective concentration (EC_50_) and a higher maximal absorbance (E_max_) (Table 2).

**Table 2:** Immunological binding activities for *Daboia russelii* snake venoms against Indian VINS Polyvalent Antivenom (VPAV).

Venom origin	EC_50_ (µg/mL)	E_max_ (Absorbance)
*Daboia russelii* (Sri Lanka)	5.895±0.043*	3.515±0.011*
*Daboia russelii* (India)	5.218±0.063*	3.797±0.044*

Values were expressed as mean ± S.E.M. from triplicate experiments. *Significant difference between venoms (p < 0.05). EC_50_: concentration of antivenom at half value of absorbance max. E_max_: maximum absorbance at the highest antivenom concentration.

### *ELISA-based immuno-profiling of D. russelii venom protein fractions*

Figure 3B and Figure 3C show the immunorecognition profile of VPAV toward the different HPLC fractions of DrSL and DrI venom proteins. All fractions were tested at a standard amount of 10 ng proteins. The immunoreactivity of VPAV was consistently low (Abs < 0.3) toward the early eluted fractions (Fractions 1-5) while increasing toward the subsequent fractions of DrSL6-10 and DrI6-7. The highest immunoreactivity was shown in fractions DrSL12-13, and DrI10-12, which were HPLC fractions that contained larger proteins with moderate to high molecular weights (Figure 1).

### *Procoagulant activities of D. russelii venoms and neutralization by antivenom*

The DrSL and DrI venoms both showed a dose-dependent procoagulant activity on human citrated plasma. The minimum coagulation doses (MCD) of the venoms were comparable at approximately 0.01 µg/mL, and the procoagulant effects were effectively neutralized by VPAV (Table 3). The MCD and ED values were not significantly different between the two venoms (p > 0.05).

**Table 3.** Procoagulant and neutralization activity of *Daboia russelii* venoms with VPAV.

Venom origin	Minimum coagulation dose (MCD) (µg/mL)	Challenge dose (2 MCD) (µg/mL)	Effective dose, ED (µg/µL)
*Daboia russelii* (Sri Lanka)	0.0115 ± 0.001	0.023	0.94 (0.87-1.03)
*Daboia russelii* (India)	0.0132 ± 0.001	0.026	1.1 (0.97-1.26)

MCD was defined as the amount of venom that caused clotting in 1 mL of citrated human plasma in 3 min. Effective dose (ED): the venom amount (µg) neutralized per (µL) of antivenom when the plasma coagulation time at 2 MCD was prolonged three times that of control (without antivenom). Results are presented as mean of triplicate ± S.E.M.

### *Anticoagulant effect of D. russelii venoms*

When assayed with APTT and PT reagents, the D. russelii venoms did not exhibit anticoagulant activity. The venoms did not prolong or increase the clotting time of the D. russelii venoms at serial concentrations when compared with the negative control (plasma with APTT/PT reagent). 

### *Lethality and neutralization of D. russelii venoms*

The intravenous median lethal doses (LD_50_) of DrSL and DrI venoms were 0.24 and 0.32 µg/g, respectively. VPAV was effective in neutralizing the venom lethality of DrSL and DrI to comparable degrees, with potency values of 3.69 mg/mL and 3.76 mg/mL (amount of venom completely neutralized per milliliter of antivenom), respectively. The in vivo neutralization parameters tested were shown in Table 4.

**Table 4.** Lethality of *Daboia russelii* venoms (Sri Lanka and Southern India) and neutralization by VPAV (Indian Polyvalent Antivenom of VINS product).

Venom	*i.v. LD*_50_ (µg/g)	ED_50_ (µL of antivenom)	ER_50_ (mg/mL)	P (mg/mL)	n-P (mg/g)
*Daboia russelii* (Sri Lanka)	0.24 (0.22-0.39)	6.25 (5.04-7.75)	4.61 (3.65-11.9)	3.69	43.4
*Daboia russelii* (India)	0.32 (0.27-0.46)	7.83 (6.40-9.58)	4.70 (3.97-6.76)	3.76	44.3

Intravenous median lethal dose (i.v. LD_50_): Venom dose in µg/g at which 50% of mice were dead. Median effective dose (ED_50_): Antivenom dose in µL at which 50% of mice survived the challenge dose of 5 x LD_50_. Median effective ratio (ER_50_): Ratio of the amount of venom (mg) to the volume dose of antivenom (mL) at which 50% of mice survived. Potency (P): Amount of venom (mg) that is completely neutralized by a unit volume (mL) of antivenom. Normalized potency (n-P): the amount of venom (mg) that is completely neutralized per unit gram of antivenom protein.

Note: values in brackets are 95% confidence interval (CI) of LD_50_, ED_50_ and ER_50_

## Discussion

The present proteomic study revealed that the venoms of D. russelii from Sri Lanka (DrSL) and Southern India (DrI) shared similar major protein families notwithstanding variations in their subtypes and relative abundances. The venom proteomes of DrSL and DrI consistently showed the dominance of phospholipase A_2_ (PLA_2_) at a strikingly high abundance of approximately 65% of total venom proteins. The PLA_2_ abundance in Russell’s viper venom has been a rather variable, disputable figure, ranging from as low as ~22% to 70% of total venom proteins in various studies. The exceptionally high abundance of PLA_2_ (>60%), which also indicated its important role in envenomation, was first reported in the venom proteome of wild-caught Pakistani Russell’s viper [12]. The high abundance observed is in contrast to the much lower values (~20 to 30%) reported previously for Russell’s vipers from other sources, including Pakistan (captive specimens in Kentucky Reptile Zoo, USA, 32.8%) [11], Mumbai (Western India, 32.5%) [15], Burdwan (Eastern India, 22.2%) and Nadia (Eastern India, 21.5%) [14], which were also investigated with chromatographic approaches. Our findings of the exceptionally high PLA_2_ abundances (current work and in Faisal et al. [12]) were, nonetheless, verified based on both chromatography (area under the curve) and gel densitometry, and were in agreement with the recent findings reported by Pla et al. [13] on specimens sourced from South India (70.6%) and Sri Lanka (60.1%). Together, the findings validated the abundance of PLA_2_ in western Russell’s viper venom from the two locales. The remarkable variations noted in the captive Pakistani Russell’s viper [11] and specimens from Western [15] and Eastern India [14] suggested possible evolutionary adaptation of the species to different ecological niches. A much lower “abundance” of PLA_2_ (23.8%) was reported in another proteomic study on a South Indian Russell’s viper venom sample [31], while it should be noted that the figure was non-quantitative (whereby the number of PLA_2_ was divided by the total number of all proteins detected), instead of relative abundance expressed in percentage of total protein weight. Meanwhile, the PLA_2_ abundance reported earlier in a Sri Lankan specimen was approximately 35% based on a semi-quantitative method of 1D SDS-PAGE [30]. The method is probably less accurate for quantification purpose compared to the sequential use of HPLC and tandem mass spectrometry applied in most snake venomic studies [48,49].

The dominance of PLA_2_ in the venom implies that the proteins play a significant pathophysiological role in Russell’s viper envenomation. Snake venom PLA_2_ can cause various complications resulted from its coagulopathic, platelet-aggregation inhibitory, cytotoxic, myotoxic, neurotoxic, pro-inflammatory and hypotensive effects [50-53]. Further examination revealed that the PLA_2_ mainly consisted of basic PLA_2_ subtypes (DrSL: ~64%; DrI: ~68%), with only a minute amount of acidic PLA_2_ in DrI (~3%) while undetected in DrSL. The basic PLA_2_ subtype VRV-PL-VIIIa (UniProt: P59071) is the major PLA_2_ identified in both DrSL (33.44%) and DrI (58.45%) venoms. VRV-PL-VIIIa is a coagulotoxin that binds to the activated factor Xa, preventing it from forming the prothrombinase complex which is crucial in hemostatic regulation [54,55]. Its catalytic action that releases arachidonic acid from membrane phospholipids also inhibits platelet aggregation, thus worsening bleeding diathesis in envenomed patients [56,57]. Besides, VRV-PL-VIIIa exhibited pre-synaptic neurotoxicity but its potency was low [58]. The proteomic finding also revealed that the basic PLA_2_ U1-viperitoxin-Dr1a or viperitoxin (UniProt: P86368), which is the primary neurotoxin implicated in Russell's Viper envenoming in Sri Lanka [59], is the second most abundant PLA_2_ subtype found in the DrSL venom, constituting 22.78% of the total venom proteins. This pre-synaptic neurotoxin, however, was present minimally in the DrI venom (3.93%). In addition, a PLA_2_ isoform homologous to the neurotoxic basic PLA_2_, DsM-S1 (UniProt: A8CG84) [60] was also present at a higher abundance in the DrSL venom compared to DrI venom. The higher overall abundance of neurotoxic PLA_2_ detected in the DrSL venom proteome corroborates pre-synaptic neurotoxicity which is more commonly reported in Sri Lankan Russell’s viper envenomation [19,20,30,61].

The SVSP family was found more abundantly in the DrSL venom proteome (approximately 2-fold higher) than DrI venom. The SVSP abundance in DrI venom is, however, comparable with that reported in another South Indian specimen [13], whereas specimens from other Indian locales (eastern and western regions) showed a more variable content of SVSP (~8% to ~14% of total venom proteins) [14,15]. On the other hand, the SVSP abundances in DrSL (current work) and the wild Pakistani Russell’s viper venom [12] were more consistent (~5.5%). The presence of diverse SVSPs including fibrinogenases and thrombin-like enzymes in DrSL and DrI venoms supports the potent procoagulant effect of the venoms, and correlates clinically with consumptive coagulopathy. Both alpha- and beta-fibrinogenases were identified in the venom proteomes; these are arginine esterases that cleave fibrinopeptides A or B from the α- and β-chains of fibrinogen to form friable microclots of fibrin. The continuous hydrolysis of fibrinogen results in hypofibrinogenemia, leading to consumptive coagulopathy and hypovolemic shock [62]. Another major SVSP detected was RVV-V gamma (UniProt: P18965), a factor V activator that selectively cleaves the Arg^1549^−Ser^1546^ linkage in the human factor V molecule [63]. Activation of factor V elicits rapid coagulation in vitro while in vivo it results in defibrinogenation and anticoagulation [64], contributing to consumptive coagulopathy in Russell’s viper envenomation.

Snake venom metalloproteinases (SVMP) are usually abundantly expressed in Asian pit viper venoms, constituting more than 30% of the proteomes [65,66]. The DrSL and DrI venoms, on the contrary, displayed low abundances of SVMP in their proteomes with remarkable intraspecific variations. The SVMP abundance in the DrSL venom (7.34%) was comparable to that reported previously for Sri Lankan specimens (4.80-6.90%) [13,30]. Marked geographical variation in SVMP, however, was observed across the various Indian specimens. The South Indian specimens had little SVMP (<4%, current work and Pla et al.) [13], whereas specimens from Eastern and Western India showed exceptionally high SVMP abundances (17.71-24.80%) [14,15]. Despite the variable protein abundance, the major SVMP reported in these Russell’s viper venom proteomes consistently belonged to the SVMP PIII subclass. Of the PIII SVMP detected, Factor X activating enzyme (RVV-X) was the most well-characterized subtype for its hemotoxic role in Russell’s viper envenomation [67]. The metalloproteinase proteolytically cleaves the target bond in the factor X molecule, while the light-chain snaclecs form a secondary binding site specific for interaction with the Gla domain of factor X, thus activating factor X and initiating blood coagulation [68]. Along with the procoagulant SVSPs, the factor X activator contributes to the exacerbation of venom-induced consumptive coagulopathy. Although PIII SVMP are typically ‘hemorrhagins’ (that induce hemorrhage through degradation of vascular basement membrane and capillaries) [69,70], no significant dermal hemorrhagic activity of the venom was noted in this study. The procoagulant nature of the major PIII SVMP (RVV-X) and its low abundance in the Russell’s viper venom shed light on the lack of local hemorrhagic effect in mice as shown in this study and a previous report [71]. The finding is also consistent with clinical observations in which Russell’s viper envenomation typically causes systemic hemorrhage rather than localized bleeding [19,20,72,73].

The abundances of KSPI were comparable between the Sri Lankan (3.03-5.35%) and South Indian (2.40-5.41%) specimens, but more variable in the specimens from Western and Eastern India (12.50-22.90%), Pakistan (16.00-28.40%) and Bangladesh (7.90%). KSPIs were known to exhibit pro-inflammatory, serine protease-inhibiting and hemotoxic (potentially anticoagulant) properties [74-76]. Other venom proteins detected were generally less than 5% per protein family in the venoms, with abundances that were more variable across the different geographical specimens. These include CRiSP, CTL (snaclecs), vNGF, LAAO, vEGF, ‘5NUC, PDE, DIS and SMI, which may serve ancillary functions such as anti-platelet, cytotoxic and proinflammatory activities.

Preceding reports showed that D. russelii envenomation in Sri Lanka and South India caused capillary leakage syndrome (CLS), a pathological condition triggered by increased vascular permeability secondary to endothelial damage. This results in edema formation, hypoalbuminemia and intravascular shock [19,77]. It was proposed that CLS is caused by the formation of lysophosphatidic-inflammatory metabolites (PLA_2_ activity), apoptosis (LAAO activity) and direct damage to the capillary basement membrane by SVMP [23], as well as the action of vEGF that increases vascular permeability [78,79]. The presence of vEGF in DrSL and DrI in the present work, together with the copious PLA_2_ and other pro-inflammatory toxins, likely contribute to the distinct pathology of CLS reported in Russell’s viper envenomation in Sri Lanka and South India.

The study also showed that DrSL and DrI venoms were highly potent in inducing plasma coagulation, consistent with the substantial amount of procoagulant SVSP and SVMP in the venom proteomes. The findings revealed that the procoagulant activity of D. russelii venoms is calcium-dependent, in line with the model proposed by Morita [68] which suggested that Ca^2+^ ions are needed to induce a conformational change in the gamma-carboxyglutamate domain of Factor X for it to be recognized by the procoagulant toxin [67]. Interestingly, anticoagulant activity was not detected in the venoms of DrSL and DrI, consistent with the study reported by Prasad et al. [71] (Southern and Western Indian D. russelii specimens). Nevertheless, a PLA_2_ isoform isolated from D. russelii venom (Pakistani origin) had been shown to possess in vitro anticoagulant activity previously [80]. The absence of in vitro anticoagulant activity in the whole venom could be explained by the potent procoagulant activity of the venom overweighing the in vitro anticoagulant effect of the venom. Clinically, it is anticipated that Russell’s viper envenomation leads to coagulopathic complication through multiple pathways including the consumption of various clotting factors by the potent procoagulant enzymes (hence, consumptive coagulopathy), and direct anticoagulant effect from anticoagulant toxins in the venom [81,82].

In mice, DrSL and DrI venoms both exhibited potent lethality (i.v. LD_50_ = ~0.2-0.3 µg/g), similar to that observed in the specimens from Pakistan and Bangladesh (~0.19 µg/g) [12,13]. Clinically, Russell’s viper envenomation in humans is extremely deadly with a fatality rate of 37.9%, as reported earlier in central India [83]. Findings from the immunoreactivity study revealed conserved protein antigenicity in both DrSL and DrI venoms, supporting the effective binding of VPAV to the venom proteins. Further immunorecognition profiling inferred that VPAV was more immunoreactive toward HPLC fractions that contained abundant PLA_2_, SVSP and SVMP. These proteins were the major components detected in the proteomes, and the main culprit toxins implicated in the pathophysiology of Russell’s viper envenomation [11,12,14,15,31]. Nevertheless, the antivenom exhibited low immunoreactivity toward early eluted fractions (Fractions 1-5) which were made up of, primarily, low molecular mass proteins. The observation is consistent with previous reports which suggested that small venom proteins are less antigenic, thus antivenom immunoreactivity is generally low toward toxins of low molecular mass [12,66,84,85].

Essentially, an effective antivenom must be able to neutralize the major toxins present in the venom, so that the principal toxicity manifested in envenomation can be mitigated or reversed. In this regard, VPAV effectively inhibited the procoagulant activity of DrSL and DrI venoms, implying that the antivenom was able to neutralize the main procoagulant venom proteins such as SVMP (e.g. FXa/RVV-X activator heavy chain) [86], SVSP (e.g. RVV-V toxins) [87] and CTL (e.g. coagulation FXa light chain) [88]. The efficacy of VPAV was further evaluated in vivo with the WHO recommended ‘gold standard’ protocol of neutralization in mice [89]. In the present work, we found that the efficacy of VPAV in neutralizing the lethality of DrI and DrSL venoms is modest, consistent with that reported for specimens from Pakistan (unspecified locale) and Sri Lanka (unspecified locale) [13,90,91]). The in vivo neutralization of Indian Russell’s viper venoms was, however, not reported previously and thus could not be compared with our current finding. These preclinical data thus far indicated that the Indian polyvalent antivenom could neutralize the lethality of Sri Lankan and Indian D. russelii venoms to different extents. 

Taken together, the neutralization findings (from the present work and previous studies) indicate that protein epitopes were largely conserved in Russell’s viper venoms sampled from various locales in South Asia. The neutralization potency of antivenom, however, may be optimized with region-specific venoms used as immunogens during antivenom production [89,92]. The approach of toxin-targeted antivenom production, where species-specific principal toxins are used as or added to the immunogen mixture, may also improve the specificity and potency of the antivenom. From the practical standpoint, assuming that an adult Indian Russell’s viper injects 100 mg venom per bite (author’s experience in venom milking), the antivenom neutralization potency of approximately 3.6 mg/mL (equivalent to 36 mg/vial) indicates that at least 3 vials of antivenom will be needed in the initial dosing regimen. In practice, additional doses are typically required as the toxins are slowly absorbed into the systemic circulation from the bite site while the antivenom is simultaneously eliminated from the body. Clinically, the recommended therapeutic dose of VPAV for patients envenomed by Russell’s viper in Sri Lanka is 10 vials of the antivenom given as an infusion in normal saline over one hour. If the coagulopathy persists (indicated by incoagulable blood on 20-minute whole blood clotting test, 20WBCT) after 6 hours, an additional 10 vials of antivenom will be administered. This underscores the need for antivenom with a higher potency to ensure that effective neutralization can be achieved with a lower dose of antivenom, which in turn will reduce the cost of treatment and the risk of hypersensitive reaction. Findings from antivenomics and toxin-fraction immunoreactivity studies should provide further insights into the improvement of antivenom production [48]. In this context, we propose that key toxins should be identified and derived from the respective geographical specimens to formulate a new immunogen recipe that can efficiently elicit neutralizing antibodies against the targeted toxins [93].

## Conclusion

The venoms of D. russelii from Sri Lanka (DrSL) and South India (DrI) showed comparable proteomic profiles, with PLA_2_ constituting the most abundant and diverse proteins. Intraspecific venom variation was minor and mainly involved proteins with ancillary functions. Both DrSL and DrI venoms were highly procoagulant and lethal, consistent with the pathophysiology of Russell’s viper envenomation. The Indian antivenom, VPAV, showed comparable binding activity toward both venoms, and modestly neutralized the procoagulant as well as the lethal effects of the venoms. It is anticipated that improving the neutralization potency of the antivenom will lead to optimal dosing, thus reducing the cost of treatment and the risk of hypersensitive reaction. This may be achieved by selectively identifying and incorporating the principal toxins of venoms from various regions into the immunogen formulation, thus producing an antivenom with pan-regional, wider geographical utility.

## Supplementary material

The following online material is available for this article:

Additional file 1. Protein assignment of Sri Lankan *Daboia russelii* (DrSL) venom by fractions of reversed-phase HPLC. Data were generated from ESI-LCMS/MC analysis of in-solution digested peptides.

Additional file 2. Protein assignment of Indian *Daboia russelii* (DrI) venom by fractions of reversed-phase HPLC. Data were generated from ESI-LCMS/MC analysis of in-solution digested peptides.

Additional file 3. Mass spectrometry information of Sri Lankan and Indian *Daboia russelii* HPLC-eluted venom fractions from nano-ESI-LCMS/MS.
