# Supplementary material for: Proteomics, toxicity and antivenom neutralization of Sri Lankan and Indian Russell’s viper (Daboia russelii) venoms
Source: J Venom Anim Toxins Incl Trop Dis. 2021 Apr 30;27:e20200177. doi: 10.1590/1678-9199-JVATITD-2020-0177 (PMC8092856; doi:10.1590/1678-9199-JVATITD-2020-0177)
Supplement: Additional file 1. [file 1678-9199-jvatitd-27-e20200177-s1.pdf]

J Venom Anim Toxins incl Trop Dis

https://doi.org/10.1590/1678-9199-JVATITD-2020-0105

Supplementary Material to "Proteomics, toxicity and antivenom neutralization of Sri Lankan and Indian Russell's viper (*Daboia russelii*) venoms"

Additional file 1. Protein assignment of Sri Lankan *Daboia russelii* (DrSL) venom by fractions of reversed-phase HPLC. Data were generated from ESI-LCMS/MC analysis of in-solution digested peptides.

Fraction	Protein designated ID	Protein name*	Database accession/ species name	Protein score	Distinct peptides matched (#)	Relative abundance (%)
1	*DrSL1*	Snake venom metalloprotease inhibitor precursor	CL1276.Contig 1_DRSL *(D. russelii)*	31.19	2	1.69%
2	*DrSL2*	Kunitz-type serine protease inhibitor 3	Q2ES48 *(D. russelii)*	84.81	4	1.43%
	DrSL1	Snake venom metalloprotease inhibitor precursor	CL1276.Contig 1_DRSL (*D. russelii*)	43.86	3	0.66%
	DrSL3	Disintegrin trigramin-gamma	P62383 *(T. gramineus)*	36.9	2	0.05%
3	*DrSL2*	Kunitz-type serine protease inhibitor 3	Q2ES48 *(D. russelii)*	140.09	8	1.54%
	DrSL4	Basic phospholipase A2 VRV-PL-VIIIa	P59071 *(D. russelii)*	109.01	6	1.06%
	DrSL5	U1-viperitoxin-Dr1a	P86368 *(D. russelii)*	92.22	5	1.56%
	DrSL6	Factor X activator heavy chain	K9JAW0 *(D. russelii)*	34.91	2	0.04%
4	*DrSL5*	U1-viperitoxin-Dr1a	P86368 *(D. russelii)*	50.46	3	0.35%
	DrSL4	Basic phospholipase A2 VRV-PL-VIIIa	P59071 *(D. russelii)*	49.59	3	0.35%
	DrSL7	Zinc metalloproteinase-disintegrin-like VLAIP-A	Q4VM08 *(M. lebetina)*	26.96	3	0.42%
	DrSL8	DSAIP	A0A2H4Z2Y9 *(D. siamensis)*	39.1	3	0.41%
	DrSL9	Venom nerve growth factor	P30894 *(D. russelii)*	28.91	2	0.90%
5	*DrSL4*	Basic phospholipase A2 VRV-PL-VIIIa	P59071 *(D. russelii)*	117.4	6	0.20%
	DrSL4	Basic phospholipase A2 VRV-PL-VIIIa	B3RFI8 *(D. russelii)*	92.43	5	0.15%
	DrSL10	Snake venom vascular endothelial growth factor toxin VR-1	P67861 *(D. russelii)*	105.67	6	2.16%
	DrSL11	Basic phospholipase A2 homolog Tpu-K49b	Q2YHJ8 *(T. puniceus)*	73.5	4	0.16%
	DrSL2	Kunitz-type serine protease inhibitor 3	Q2ES48 *(D. russelii)*	39.66	2	0.06%
	DrSL9	Venom nerve growth factor	P30894 *(D. russelii)*	38.69	2	0.08%
6	*DrSL4*	Basic phospholipase A2 VRV-PL-VIIIa	P59071 *(D. russelii)*	266.23	14	16.50%

Fraction	Protein designated ID	Protein name*	Database accession/ species name	Protein score	Distinct peptides matched (#)	Relative abundance (%)
	DrSL5	U1-viperitoxin-Dr1a	P86368 *(D. russelii)*	97.79	5	10.92%
	DrSL12	Cysteine-rich secretory protein Dr-CRPK	CL3658.Contig 2_DRSL *(D. russelii)*	61.64	4	4.92%
	DrSL13	Cysteine rich secretory protein	ASU45033 *(D. russelii)*	32.19	2	1.05%
	DrSL14	Beta-fibrinogenase-like	E5L0E4 *(D. siamensis)*	30.93	2	0.0007%
	DrSL15	Venom serine proteinase-like protein 2	Q9PT40 *(M. lebetina)*	30.49	2	1.09%
7	*DrSL16*	RVV-V gamma-like protein precursor	P18965 *(D. siamensis)*	87.4	9	0.64%
	DrSL16	RVV-V gamma-like protein precursor	P18965 *(D. siamensis)*	125.6	8	1.30%
	DrSL17	Serine beta-fibrinogenase-like protein precursor	CL2958.Contig 1_DRSL *(D. russelii)*	69.44	4	0.38%
	DrSL17	Serine beta-fibrinogenase-like protein precursor	CL2958.Contig 7_DRSL *(D. russelii)*	103.42	7	1.48%
	DrSL5	U1-viperitoxin-Dr1a	P86368 *(D. russelii)*	117.19	7	0.79%
	DrSL4	Basic phospholipase A2 VRV-PL-VIIIa	P59071 *(D. russelii)*	98.32	6	1.89%
	DrSL18	Serine alpha-fibrinogenase-like protein precursor	E5L0E3 *(D. siamensis)*	52.66	3	0.18%
	DrSL19	L-amino-acid oxidase	G8XQX1 *(D. russelii)*	44.69	3	0.04%
	DrSL20	L-amino-acid oxidase	CL1662.Contig 1_DRSL *(D. russelii)*	42.69	3	0.04%
	DrSL15	Venom serine proteinase-like protein 2	Q9PT40 *(M. lebetina)*	29.76	2	0.11%
8	*DrSL4*	Basic phospholipase A2 VRV-PL-VIIIa	P59071 *(D. russelii)*	174.19	10	1.01%
	DrSL21	Basic phospholipase A2 DsM-S1	A8CG84 *(D. siamensis)*	168.55	9	0.99%
	DrSL5	U1-viperitoxin-Dr1a	P86368 *(D. russelii)*	160.33	9	0.84%
	DrSL17	Serine beta-fibrinogenase-like protein precursor	CL2958.Contig 7_DRSL *(D. russelii)*	65.14	4	0.11%
	DrSL22	Serine beta-fibrinogenase-like protein precursor	E5L0E4 *(D. siamensis)*	49.06	3	0.11%
9	*DrSL19*	L-amino-acid oxidase	G8XQX1 *(D. russelii)*	31.29	2	0.02%
	DrSL4	Basic phospholipase A2 VRV-PL-VIIIa	P59071 *(D. russelii)*	158.2	9	5.89%
10	*DrSL5*	U1-viperitoxin-Dr1a	P86368 *(D. russelii)*	157.03	8	1.17%
	DrSL23	Factor X activator heavy chain	Unigene32626 _DRSL *(D. russelii)*	199.63	11	0.25%
	DrSL5	U1-viperitoxin-Dr1a	P86368 *(D. russelii)*	173.02	9	6.25%
	DrSL21	Basic phospholipase A2 DsM-S1	A8CG84 *(D. siamensis)*	158.63	8	6.27%

Fraction	Protein designated ID	Protein name*	Database accession/ species name	Protein score	Distinct peptides matched (#)	Relative abundance (%)
	DrSL4	Basic phospholipase A2 VRV-PL-VIIIa	P59071 *(D. russelii)*	156.01	8	5.25%
	DrSL24	Dabocetin alpha subunit	K9JBU0 *(D. russelii)*	58.22	4	0.13%
	DrSL17	Serine beta-fibrinogenase-like protein precursor	CL2958.Contig 7_DRSL *(D. russelii)*	25.55	2	0.06%
11	*DrSL23*	Factor X activator heavy chain	Unigene32626 _DRSL *(D. russelii)*	279.41	15	0.47%
	DrSL4	Basic phospholipase A2 VRV-PL-VIIIa	P59071 *(D. russelii)*	128.36	7	0.45%
	DrSL5	U1-viperitoxin-Dr1a	P86368 *(D. russelii)*	102.47	5	0.41%
	DrSL24	Dabocetin alpha subunit	K9JBU0 *(D. russelii)*	93.84	6	0.23%
	DrSL25	Dabocetin beta subunit	Unigene3920_ DRSL *(D. russelii)*	64.3	5	0.47%
	DrSL26	C-type lectin A12	Unigene30367 _DRSL *(D. russelii)*	62.98	4	0.09%
	DrSL27	Coagulation factor X activating enzyme light chain	Q4PRD1 *(D. russelii)*	40.19	2	0.09%
	DrSL28	Factor X activator light chain 2	Q4PRD2 *(D. siamensis)*	35.48	2	0.23%
	DrSL17	Serine beta-fibrinogenase-like protein precursor	CL2958.Contig 7_DRSL *(D. russelii)*	35.06	2	0.02%
	DrSL29	L-amino acid oxidase	CL4302.Contig 1_TA *(T. albolabris)*	28.17	2	0.05%
	DrSL30	5'-nucleotidase	W8EFS0 *(M. lebetina)*	27.71	2	0.01%
12	*DrSL23*	Factor X activator heavy chain	Unigene32626 _DRSL *(D. russelii)*	289.75	16	0.64%
	DrSL31	Zinc metalloproteinase-disintegrin HV1	Unigene3635 *(T. purpureomaculatus)*	48.53	3	0.24%
	DrSL21	Basic phospholipase A2 DsM-S1	A8CG84 *(D. siamensis)*	174.82	11	0.29%
	DrSL4	Basic phospholipase A2 VRV-PL-VIIIa	P59071 *(D. russelii)*	167.76	10	0.30%
	DrSL5	U1-viperitoxin-Dr1a	P86368 *(D. russelii)*	97.87	5	0.24%
	DrSL32	Snaclec dabocetin subunit alpha	Q38L02 *(D. siamensis)*	68.06	6	0.14%
	DrSL28	Factor X activator light chain 2	Q4PRD2 *(D. siamensis)*	52.33	3	0.45%
	DrSL27	Coagulation factor X activating enzyme light chain	Q4PRD1 *(D. russelii)*	40.97	2	0.34%
	DrSL26	C-type lectin A12	Unigene30367 _DRSL *(D. russelii)*	34.24	2	0.07%
	DrSL29	L-amino acid oxidase	CL4302.Contig 1_TA *(T. albolabris)*	32.62	2	0.04%

Fraction	Protein designated ID	Protein name*	Database accession/ species name	Protein score	Distinct peptides matched (#)	Relative abundance (%)
	DrSL25	Dabocetin beta subunit	Unigene920_DRSL *(D. russelii)*	31.97	2	0.15%
13	*DrSL23*	Factor X activator heavy chain	Unigene32626_DRSL *(D. russelii)*	212.98	12	1.19%
	DrSL31	Zinc metalloproteinase-disintegrin HV1	Unigene3635_TP *(T. purpureomaculatus)*	96.26	6	0.95%
	DrSL33	Zinc metalloproteinase-disintegrin ACLD	CL9202.Contig4_TA *(T. albolabris)*	40.38	2	0.24%
	DrSL34	Zinc metalloproteinase-disintegrin-like VMP-III	C9E1S0 *(A. leucostoma)*	33.92	2	2.19%
	DrSL30	5'-nucleotidase	W8EFS0 *(M. lebetina)*	202.4	12	0.43%
	DrSL35	Phosphodiesterase 1	CL3655.Contig2_CA *(C. adamanteus)*	130.36	8	0.21%
	DrSL20	L-amino-acid oxidase	CL1662.Contig1_DRSL *(D. russelii)*	124.06	8	0.85%
	DrSL36	L-amino-acid oxidase	Q4F867 *(D. siamensis)*	83.52	5	1.22%
	DrSL37	Chain A, Amine Oxidase	Q6TGQ8 *(B. moojeni)*	32.53	2	0.60%
	DrSL4	Basic phospholipase A2 VRV-PL-VIIIa	P59071 *(D. russelii)*	68.52	5	0.41%
	DrSL27	Coagulation factor X activating enzyme light chain, RVV-X-light chain, LC1=metalloproteinase with disintegrin-like and C-type lectin-like domains	Q4PRD1 *(D. russelii)*	35.72	2	0.40%
	DrSL38	Snaclec 4	Q4PRC9 *(D. siamensis)*	30.29	2	0.26%
	DrSL5	U1-viperitoxin-Dr1a	P86368 *(D. siamensis)*	33.98	2	0.26%
	DrSL39	P68 alpha subunit	K9JDF2 *(D. siamensis)*	30.63	2	0.13%
	DrSL28	Factor X activator light chain 2	Q4PRD2 *(D. siamensis)*	28.83	2	2.05%
	DrSL40	xaa-Pro aminopeptidase 2	Unigene19296_EC *(E. carinatus)*	28.31	2	0.05%
	DrSL41	Zinc metalloproteinase-disintegrin stejnihagin-B	CL4568.Contig1_TP *(T. purpureomaculatus)*	26.79	2	0.13%
	DrSL42	Zinc metalloproteinase-disintegrin jerdonitin	A0A194ARM6 *(P. mucrosquamatus)*	25.04	2	0.14%

B. moojeni: *Bothrops moojeni*; *C. adamanteus*: *Crotalus adamanteus*; *D. russelii*: *Daboia russelii*; *D. siamensis*: *Daboia siamensis*; *E. carinatus*: *Echis carinatus*; *M. lebetina*: *Macrovipera lebetina*; *P. mucrosquamatus*: *Protobothrops mucrosquamatus*; *T. albolabris*: *Trimeresurus albolabris*; *T. gramineus*: *Trimeresurus gramineus*; *T. puniceus*: *Trimeresurus puniceus*; *T. purpureomaculatus*: *Trimeresurus purpureomaculatus.*
*Protein names taken from protein sequence database based on homology match in BLAST and UniProt search.
Peptide sequences and mass/charge ratios of all corresponding proteins are shown in Additional file 3.
#Number of distinct peptides matched to identified proteins.
