# Supplementary material for: Proteomics, toxicity and antivenom neutralization of Sri Lankan and Indian Russell’s viper (Daboia russelii) venoms
Source: J Venom Anim Toxins Incl Trop Dis. 2021 Apr 30;27:e20200177. doi: 10.1590/1678-9199-JVATITD-2020-0177 (PMC8092856; doi:10.1590/1678-9199-JVATITD-2020-0177)
Supplement: Additional file 2. [file 1678-9199-jvatitd-27-e20200177-s2.pdf]

J Venom Anim Toxins incl Trop Dis

https://doi.org/10.1590/1678-9199-JVATITD-2020-0105

Supplementary Material to "Proteomics, toxicity and antivenom neutralization of Sri Lankan and Indian Russell's viper (*Daboia russelii*) venoms"

Additional file 2. Protein assignment of Indian *Daboia russelii* (DrI) venom by fractions of reversed-phase HPLC. Data were generated from ESI-LCMS/MC analysis of in-solution digested peptides.

Fraction	Protein designated ID	Protein name*	Database accession/ species name	Protein score	Distinct peptides matched (#)	Relative abundance (%)
1	*DrI1*	Kunitz-type protease inhibitor	H9BFA3 *(D. russelii)*	36.21	2	2.69%
	DrI2	Disintegrin jerdostatin	Q7ZZM2 *(P. jerdonii)*	33.13	2	0.32%
2	*DrI3*	Kunitz-type serine protease inhibitor B5	A8Y7P5 *(D. siamensis)*	74.66	4	0.82%
	DrI1	Kunitz-type protease inhibitor	H9BFA3 *(D. russelii)*	63.84	3	1.82%
	DrI4	Basic phospholipase A2 VRV-PL-VIIIa	P59071 *(D. russelii)*	44.82	3	0.12%
3	*DrI5*	DSAIP	A0A2H4Z2X4 *(D. siamensis)*	105.35	6	0.05%
	DrI6	Venom nerve growth factor	P30894 *(D. russelii)*	65.08	4	0.05%
	DrI7	U1-viperitoxin-Dr1a	P86368 *(D. russelii)*	58.98	3	0.17%
	DrI4	Basic phospholipase A2 VRV-PL-VIIIa	P59071 *(D. russelii)*	53.59	3	0.17%
	DrI1	Kunitz-type protease inhibitor	H9BFA3 *(D. russelii)*	51.22	3	0.03%
4	*DrI5*	DSAIP	A0A2H4Z2X4 *(D. siamensis)*	110.67	6	0.14%
	DrI6	Venom nerve growth factor	P30894 *(D. russelii)*	94.43	6	0.61%
	DrI7	U1-viperitoxin-Dr1a	P86368 *(D. russelii)*	55.94	3	0.16%
	DrI8	Kunitz-type serine protease inhibitor C4	A8Y7N7 *(D. siamensis)*	37.41	2	0.06%
	DrI9	L-amino-acid oxidase	G8XQX1 *(D. russelii)*	34.06	2	0.04%
5	*DrI10*	Snake venom vascular endothelial growth factor toxin VR-1	P67861 *(D. russelii)*	62.27	4	1.21%
	DrI11	Snake venom vascular endothelial growth factor toxin OS	Unigene30051_DRSL *(D. russelii)*	60.28	4	0.31%
	DrI6	Venom nerve growth factor	P30894 *(D. russelii)*	59.86	3	1.02%
	DrI4	Basic phospholipase A2 VRV-PL-VIIIa	P59071 *(D. russelii)*	52.57	3	0.06%

Fraction	Protein designated ID	Protein name*	Database accession/ species name	Protein score	Distinct peptides matched (#)	Relative abundance (%)
6	*DrI4*	Basic phospholipase A2 VRV-PL-VIIIa	P59071 *(D. russelii)*	270.65	13	21.35%
	DrI7	U1-viperitoxin-Dr1a	P86368 *(D. russelii)*	197.49	11	1.76%
	DrI12	Beta-fibrinogenase-like	E5L0E4 *(D. siamensis)*	76.87	5	5.49%
	DrI13	Cysteine-rich secretory protein Dr-CRPK	CL3658.Contig2_DRSL *(D. russelii)*	62.11	4	1.91%
	DrI14	Venom serine proteinase-like protein 2	Q9PT40 *(M. lebetina)*	49.74	2	2.69%
	DrI15	Acidic phospholipase A2 RV-7	P31100 *(D. siamensis)*	42.59	2	3.01%
	DrI16	Basic phospholipase A2 RVV-VD	P81458 *(D. russelii)*	30.85	2	0.99%
7	*DrI17*	Cysteine-rich secretory protein Dr-CRPK	F2Q6F2 *(D. russelii)*	195.2	11	0.89%
	DrI13	Cysteine-rich secretory protein Dr-CRPK	CL3658.Contig2_DRSL *(D. russelii)*	187.5	11	0.85%
	DrI18	Cysteine-rich secretory protein Da-CRPa	F2Q6G0 *(D. acutus)*	133.34	6	0.98%
	DrI19	Cysteine-rich secretory protein Pg-CRP	F2Q6F6 *(C. godmani)*	36.36	2	0.23%
	DrI14	Venom serine proteinase-like protein 2	Q9PT40 *(M. lebetina)*	76.03	5	0.28%
	DrI12	Beta-fibrinogenase-like	E5L0E4 *(D. siamensis)*	64.33	4	0.47%
	DrI4	Basic phospholipase A2 VRV-PL-VIIIa	P59071 *(D. russelii)*	153.85	8	0.88%
	DrI4	Basic phospholipase A2 VRV-PL-VIIIa	P59071 *(D. russelii)*	133.58	7	0.63%
	DrI20	Basic phospholipase A2 DsM-S1	A8CG84 *(D. siamensis)*	132.6	7	0.94%
	DrI7	U1-viperitoxin-Dr1a	P86368 *(D. russelii)*	70.77	4	1.62%
8	*DrI21*	Factor V activator RVV-V gamma	P18965 *(D. siamensis)*	231.54	15	0.23%
	DrI12	Beta-fibrinogenase-like	E5L0E4 *(D. siamensis)*	70.84	5	0.28%
	DrI22	Serine beta-fibrinogenase-like protein precursor	CL2958.Contig2_DRSL *(D. russelii)*	64.65	4	0.03%
	DrI23	Thrombin-like enzyme	Q98TT5 *(D. acutus)*	27.01	2	0.01%
	DrI4	Basic phospholipase A2 VRV-PL-VIIIa	P59071 *(D. russelii)*	216.86	12	0.40%
	DrI4	Basic phospholipase A2 VRV-PL-VIIIa	P59071 *(D. russelii)*	188.63	10	0.24%
	DrI7	U1-viperitoxin-Dr1a	P86368 *(D. russelii)*	104.59	5	0.12%
	DrI17	Cysteine-rich secretory protein Dr-CRPK	F2Q6F2 *(D. russelii)*	126.16	8	0.03%
	DrI13	Cysteine-rich secretory protein Dr-CRPK	CL3658.Contig2_DRSL *(D. russelii)*	107.49	7	0.03%
	DrI24	Cysteine-rich secretory protein Dr-CRPB	F2Q6F3 *(D. russelii)*	34.11	2	0.04%

Fraction	Protein designated ID	Protein name*	Database accession/ species name	Protein score	Distinct peptides matched (#)	Relative abundance (%)
	DrI19	Cysteine-rich secretory protein Pg-CRP	F2Q6F6 *(C. godmani)*	26.88	2	0.01%
	DrI15	Acidic phospholipase A2 RV-7	B3RFI6 *(D. siamensis)*	104.66	5	0.13%
	DrI14	Venom serine proteinase-like protein 2	Q9PT40 *(M. lebetina)*	90.68	5	0.02%
	DrI25	Serine protease VLSP-3	E0Y420 *(M. lebetina)*	59.9	4	0.03%
	DrI26	Beta-fibrinogenase	E0Y419 *(M. lebetina)*	48.23	3	0.03%
9	*DrI27*	Serine protease VLSP-1	CL2958.Contig2_DRSL *(D. russelii)*	98.7	4	1.20%
	DrI4	Basic phospholipase A2 VRV-PL-VIIIa	P59071 *(D. russelii)*	157.24	8	0.24%
	DrI7	U1-viperitoxin-Dr1a	P86368 *(D. russelii)*	106.22	6	0.10%
	DrI28	Alpha-fibrinogenase-like	E5L0E3 *(D. siamensis)*	140.35	9	0.49%
	DrI29	Serine alpha-fibrinogenase-like protein precursor Daboia-russelii	E5L0E3 *(D. siamensis)*	94.96	6	0.36%
	DrI12	Beta-fibrinogenase-like	E5L0E4 *(D. siamensis)*	30.56	2	0.14%
	DrI30	Serine protease	CL2791.Contig2_EC *(E. carinatus)*	27.92	2	0.09%
	DrI10	Snake venom vascular endothelial growth factor toxin VR-1	P67861 *(D. russelii)*	29.83	2	0.02%
	DrI16	Basic phospholipase A2 RVV-VD	P81458 *(D. russelii)*	28.42	2	0.04%
10	*DrI4*	Basic phospholipase A2 VRV-PL-VIIIa	P59071 *(D. russelii)*	71.35	4	1.35%
11	*DrI4*	Basic phospholipase A2 VRV-PL-VIIIa	P59071 *(D. russelii)*	38.99	2	29.99%
12	*DrI4*	Basic phospholipase A2 VRV-PL-VIIIa	P59071 *(D. russelii)*	61.72	4	3.03%
	DrI31	Moderate to high molecular weight proteins	–	–	–	6.47%

C. godmani: Cerrophidion godmani; D. russelii: Daboia russelii; D. siamensis: Daboia siamensis; D. acutus: Deinagkistrodon acutus; E. carinatus: Echis carinatus; M. lebetina: Macrovipera lebetina; P. jerdonii: Protobothrops jerdonii.

*Protein names taken from protein sequence database based on homology match in BLAST and UniProt search. Peptide sequences and mass/charge ratios of all corresponding proteins are shown in Additional file 3.

#Number of distinct peptides matched to identified proteins.
